# There is no evidence that vitamin D supplementation drives the progression of Alzheimer's disease

**DOI:** 10.1111/acel.13758

**Published:** 2022-12-19

**Authors:** Adrian F. Gombart, Alexander J. Michels, Manfred Eggersdorfer

**Affiliations:** ^1^ Department of Biochemistry and Biophysics, Linus Pauling Institute Oregon State University Corvallis Oregon USA; ^2^ Linus Pauling Institute Oregon State University Corvallis Oregon USA; ^3^ Department of Internal Medicine University Medical Center Groningen Groningen The Netherlands

If there is anything the last few decades have taught us, it is that researchers must properly conduct studies on vitamin supplements before they can make health claims. In a recent paper by Lai et al. (2022), the authors state, “vitamin D supplementation worsens Alzheimer's progression” without sufficient evidence to support these conclusions. While certain findings are scientifically interesting, readers must understand substantial limitations in the approach and methodology utilized in the paper limit conclusions one can make about the role of vitamin D in brain health and Alzheimer's disease (AD).

Before proceeding further, it is important for readers to understand that there are three different forms of vitamin D pertinent to this discussion. Cholecalciferol is the form of vitamin D_3_ synthesized in the skin upon exposure to UVB rays that typically come from sunlight. It is also the form found in dietary supplements. The liver converts cholecalciferol to calcidiol (25‐hydroxyvitamin D_3_), the circulating form used by clinicians to determine vitamin D status. Certain cells in the body will then convert calcidiol to calcitriol (1,25‐dihydroxyvitamin D_3_), the “active” form of vitamin D in the body (Bikle, 2000).

Calcitriol is a high‐affinity, potent ligand for the vitamin D receptor (VDR) and regulates most VDR‐dependent gene transcription. The body only produces calcitriol under certain physiological conditions, including low serum calcium levels or immune activation, and tightly controls its synthesis and catabolism to limit its physiological effects. On the other hand, cholecalciferol and calcidiol are considered inactive forms of vitamin D_3_, as they are low‐affinity ligands for VDR (Bikle, 2000). Because these three forms are not functionally or physiologically equivalent, referring to them all as “vitamin D” creates a great deal of confusion, especially since cholecalciferol is found in vitamin D supplements or fortified foods and beverages, but calcitriol is appropriately classified as a drug (Vieth, 2020).

The study by Lai et al. begins with data from neuronal cell culture. Here, the authors demonstrate that treatment with both calcidiol and calcitriol increases apoptosis and autophagy in undifferentiated SH‐SY5Y cells upon exposure to Aβ42. SH‐SY5Y is a neuroblastoma cell line typically used in experiments after differentiation into a homogeneous, more mature neuron‐like cell, making them useful for AD research (Agholme et al., 2019). Prior reports show calcitriol protects differentiated SH‐SY5Y cells against Aβ(1–42) peptide cytotoxicity (Vieth, 2020), although others have reported protection of undifferentiated SH‐SY5Y cells from Aβ(25–35) toxicity upon treatment with calcitriol (Lin, Chang, et al., 2020). While the study by Lai et al. reports contradictory findings, the authors do not discuss them in the context of the greater literature base, so it is unclear what could have explained these divergent responses.

Encouraged by their cell culture findings, the authors performed a vitamin D (cholecalciferol) feeding study in an APP/PS1 transgenic mouse model. This model, like most murine models for AD, recapitulates a rare form of AD that mirrors limited aspects of human AD pathology. It does not represent normal cognitive decline or even commonly seen forms of AD in humans. These animals also have difficulties maintaining vitamin D status, as evidenced by lower serum calcidiol levels compared to wild‐type controls when maintained on a 600 IU/kg cholecalciferol diet. Feeding the mice a high vitamin D diet containing 8044 IU/kg cholecalciferol starting at the age of 4.5 months restored serum calcidiol levels. However, after 3 months of supplementation, the authors observed “more severe Aβ plaque deposits and reactive gliosis in the hippocampus compared to the controls,” an increased expression of pro‐degenerative factors in the hippocampus, and worse cognitive functioning in the Morris water maze.

These findings are at odds with prior results in which vitamin D supplementation or treatment with calcitriol in several other mouse models of AD reduced amyloid burden and improved cognition (Durk et al., 2014; Lin, Lin, et al., 2020; Morello et al., 2018; Yu et al., 2011). In the 5XFAD mouse model, for example, female mice fed a high vitamin D (as cholecalciferol) diet (7500 IU/kg) had fewer plaques in the frontal cortex, hippocampus and neocortex, decreased astrogliosis, and cognitive defects as compared to the untreated mice (Landel et al., 2016). Researchers also observed similar effects of vitamin D supplementation in the AβPP (Durk et al., 2014) and the 3xTg‐AD (Landel et al., 2016) transgenic mouse models. In Tg2576 and TgCRND8 mice, calcitriol administration decreased plaque burden and improved cognitive function (Lin, Lin, et al., 2020). Furthermore, intraperitoneal injection of calcitriol also protected Long‐Evans rats from neuronal degeneration after intrahippocampal injection of aggregated Aβ(1–42; Pierucci et al., 2017). Again, the authors do not adequately discuss their findings in the context of the prior literature. The findings from this and prior studies underscore the importance of using multiple animal models to evaluate potential therapies and translate findings to humans (Drummond & Wisniewski, 2017).

The second half of the paper is a retrospective, population‐based longitudinal study that attempts to quantify the risk for dementia and mortality in older adults taking vitamin D_3_ supplements. According to the authors, their analysis indicates prolonged supplementation worsens the progression of AD by increasing dementia and mortality risk. However, this is a completely inaccurate interpretation of the data. While described as “taking vitamin D_3_ supplements during the period 2000‐2009”, the participant data comes from Taiwan's National Health Insurance Research Database that describes the use of calcitriol, a prescription medication—not a dietary supplement. Physicians prescribe calcitriol to increase calcium levels in patients whose kidneys or parathyroid glands are not functioning normally. A prescription for calcitriol is not indicative of good health, as the individuals taking calcitriol often have one or more comorbidities, such as osteoporosis, thyroid disorders, diabetes, hyperlipidemia, and hypertension. Each of these comorbidities indicates a predisposition toward dementia. Calcitriol is also often prescribed to patients with chronic kidney disease, who are already prone to cognitive decline and AD (Zhang et al., 2020). Considering the potent nature of calcitriol, it cannot be equated to taking vitamin D_3_ as a dietary supplement.

Of note, calcitriol overuse is associated with an increased risk of hypercalcemia. Hypercalcemia has been associated with dementia and changes in cognition (de Oliveira Martins Duarte et al., 2019; Lourida et al., 2015; Walker & Silverberg, 2018). Prolonged elevation in serum calcium levels, even if not to the level of hypercalcemia, can predict cognitive decline and conversion from nondemented status to AD (Ma et al., 2021). Since Lai et al. found that only medium‐to‐long‐term calcitriol use (but not short‐term use) was associated with increased dementia or mortality risk, it is possible what the authors reported were side effects of a prolonged drug regimen, not the physiological effects associated with vitamin supplementation.

In contrast to the authors' conclusions, the current body of evidence from human clinical studies supports the role of vitamin D in cognitive health. Observational research shows that a higher 25(OH)D_3_ (calcidiol) status is associated with a lower risk for AD and all‐cause dementia (DeLuca et al., 2013; Littlejohns et al., 2014; Mayne & Burne, 2019). Randomized controlled trials with vitamin D_3_ (cholecalciferol) supplementation have reported improved AD bio‐measures and cognitive function (Jia et al., 2019). In older adults, vitamin D_3_ supplementation either improved or had no association with declines in cognitive function (Kang et al., 2021; Navale et al., 2022; Rossom et al., 2012; Tong et al., 2020).

While there are clearly opportunities for more research on the role of vitamin D supplementation in cognitive health, the authors' conclusions that “vitamin D supplementation worsens Alzheimer's progression” is not justified. Conflating the data from cell culture, a murine model system and a retrospective epidemiological observation using prescription calcitriol reaches this flawed conclusion and lies in stark contrast to the large body of evidence that vitamin D status and vitamin D supplementation support cognitive health. The findings of this paper should have been placed in the proper context before these inflammatory statements on AD were released for public view.

## AUTHOR CONTRIBUTIONS

All authors designed the manuscript and read and approved the final manuscript.

## FUNDING INFORMATION

The development of the paper received no external funding.

## CONFLICT OF INTEREST

AFG has received research funding from Bayer Consumer Care and has acted as an advisor/consultant for and has received reimbursement for travel and/or speaking from Bayer Consumer Care, GSK, DSM, Kellogg's, and The Coca‐Cola Company. AJM consults for dietary supplement companies. ME is president of the Gesellschaft für Angewandte Vitaminforschung (Germany), is a member of the scientific board of PM International, and consults for companies in the nutrition and supplement field.

## Data Availability

None.
